# Improved PTV coverage and OAR sparing with stereotactic MRI-guided online adaptive radiotherapy with elective fields in pancreatic cancer

**DOI:** 10.1016/j.tipsro.2025.100354

**Published:** 2025-11-15

**Authors:** Dinah Konnerth, Hidehiro Hojo, Frederik Fuchs, Mohamed A. Shouman, Diana-Coralia Dehelean, Aurélie Gaasch, Franziska Walter, Chukwuka Eze, Sebastian N. Marschner, Sina Mansoorian, Sebastian H. Maier, Vanessa da Silva Mendes, Jan Hofmaier, Maximilian Niyazi, Claus Belka, Stefanie Corradini, Paul Rogowski

**Affiliations:** a Department of Radiation Oncology, University Hospital LMU, Munich, Germany; b Department of Radiation Oncology and Particle Therapy, National Cancer Center Hospital East, Kashiwa, Japan; c Bavarian Cancer Research Center (BZKF), Munich, Germany; d German Cancer Consortium (DKTK), Partner Site Munich, Munich, Germany; e Department of Radiation Oncology, University Hospital Tübingen, Tübingen, Germany; f German Cancer Consortium (DKTK), Partner Site Tübingen, Germany

**Keywords:** Pancreatic cancer, SBRT, SMART, Dosimetry, OAR, Online adaptation

## Abstract

**Purpose:**

Ablative stereotactic radiotherapy for pancreatic ductal adenocarcinoma (PDAC) is limited by adjacent radiosensitive organs at risk (OAR) that exhibit significant daily positional variability. This study aims to investigate the benefit of stereotactic MRI-guided online adaptive radiotherapy (SMART) with elective fields on target coverage and OAR dose sparing in patients with PDAC.

**Methods:**

We retrospectively analysed 62 consecutive PDAC patients treated with SMART between 2020–2024, totaling 310 fractions. For each fraction, a non-adapted plan (PLAN_PREDICT_) was compared to its online-adapted counterpart (PLAN_REOPTIMIZED_). We assessed planning target volume (PTV) coverage and OAR constraints. Plan-adaptation was classified as “useful” when the PLAN_REOPTIMIZED_ met all objectives or improved PTV coverage or OAR sparing by ≥ 10 %, as “futile” when PLAN_REOPTIMIZED_ failed to meet or markedly improve objectives, and as “not necessary” when PLAN_PREDICT_ already met all objectives.

**Results:**

Prescribed doses were 40 Gy (71 %) and 33 Gy (29 %) over five fractions. A simultaneous integrated boost approach was used in 47 % of patients. The PLAN_PREDICT_ met all planning goals in 1 % of fractions, which increased to 72 % with PLAN_REOPTIMIZED_. Adaptation yielded significant improvements in PTV coverage (p < 0.001) and reductions in V33Gy for stomach (median 0.26 cc vs. 0.01 cc), duodenum (median 1.08 cc vs. 0.07 cc) and bowel (median 2.04 cc vs. 0.06 cc), all p < 0.001. Adaptation was deemed useful in 305 (98 %), not necessary in 4 (1 %) and futile in 1 fraction (0.3 %).

**Conclusion:**

For the vast majority of PDAC patients treated with stereotactic radiotherapy with elective fields daily online adaptation enhances target coverage and OAR sparing.

## Highlights

• Online adaptation increased the proportion of fractions meeting all PTV coverage and OAR constraints from 1 % to 72 %.
• Adaptation produced significant gains in PTV coverage and substantially reduced V_33Gy_ to stomach, duodenum, and bowel.
• Adaptation “useful” in 98 % of fractions, “not necessary” in 1 %, and “futile” in 0.3 %, highlighting its benefit in stereotactic MRIgART with elective target volumes for PDAC.

## Graphical abstract

**Figure:**
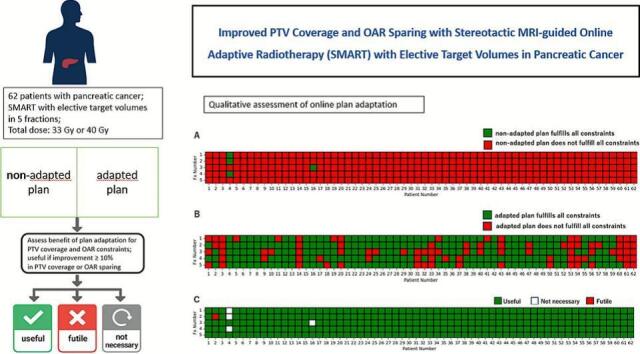

## Background

Pancreatic ductal adenocarcinoma (PDAC) remains a highly aggressive malignancy, with a 5-year overall survival (OS) rate of approximately 12 % across all stages [1]. Surgical resection is the sole curative treatment. However, late detection in locally advanced or metastatic stages frequently require alternative therapy strategies. Multi-agent chemotherapy has improved outcomes in this setting [2,3], while the role of radiotherapy remains controversial in locally advanced, and, even more so, in metastatic disease. Chemoradiotherapy (CRT) delivered over 5–6 weeks is still the most common regimen [4], but recent phase 3 trials have shown limited impact on OS despite gains in local control and R0 resection rates [5,6,7] Table 1..

**Table 1:** Patient and treatment characteristics.

**Sex, n (%)**	
Female	25 (40 %)
Male	37 (60 %)
**Age, median (range)**	68.5 years, (43–89 years)
**Tumor stage, n (%) #**	
Metastatic Disease	25 (40 %)
LAPC	17 (27 %)
Local Recurrence	*15 (24 %)
Medically inoperable	5 (8 %)
**Tumor location, n (%)**	
Pancreatic head	27 (44 %)
Overlapping head - body	2 (3 %)
Pancreatic body	10 (16 %)
Overlapping body-tail	5 (8 %)
Pancreatic tail	2 (3 %)
Local Recurrence	*16 (26 %)
**Pretreatment Chemotherapy, n (%) #**	
No	19 (31 %)
Yes	43 (69 %)
**Dose concept**	
33 Gy, single dose level	12 (19 %)
40 Gy, single dose level	21 (34 %)
33 Gy / SIB 40 Gy	29 (47 %)
**Target volumes, median (range)**	
GTV	37 cc (7–162 cc)
CTV	214 cc (81–676 cc)
PTV__OPT_	216 cc (39–673 cc)
PTV_elect__OPT_	210 cc (132–662 cc)
PTV_SIB__OPT_	57 cc (18–142 cc)
**Treatment time, median (range, interquartil range)**	70 min (38 min – 131 min, 62–80 min)

*One metastatic patient was treated for a metachronous local recurrence. He was categorized as metastatic, however, regarding the tumor location he was counted as locally recurrent. #Sum of percentages less than 100 % due to rounding. Abbreviations: GTV: gross tumor volume; Gy: Gray; LAPC: locally advance pancreatic adenocarcinoma; PTV__OPT_: optimized planning target volume; PTV_elect__OPT_: optimized elective planning target volume; PTV_SIB__OPT_: optimized simultaneous integrated boost planning target volume; SIB: simultaneous integrated boost

A major limitation of CRT is balancing adequate tumor dosing with the risk of toxicity. Stereotactic body radiation therapy (SBRT) has emerged as a strategy to deliver higher biological effective doses (BED) to smaller treatment volumes, potentially improving outcomes and reducing toxicity [8]. However, anatomical proximity to critical gastrointestinal luminal organs at risk (OAR) and their motion complicates precise delivery [9]. Traditional SBRT protocols, which depend on planning CT scans and daily low-resolution image guidance, can only compensate for interfractional anatomical changes to a limited extent, while intrafractional changes are usually only estimated using surrogate markers like the body surface. To address these uncertainties, safety margins are typically applied or an ITV approach is followed [10]. However, increasing the margins around the tumor to ensure accurate target coverage also increases the risk of added toxicity to nearby OARs. On the other hand, expanding safety margins around OARs through the creation of planning risk volumes (PRV), which are subtracted from the planning target volume (PTV), can compromise target volume coverage.

Stereotactic MRI-guided online adaptive radiotherapy (SMART) addresses these limitations by integrating magnetic resonance imaging (MRI) with a linear accelerator, offering superior soft-tissue visualization and daily adaptive replanning. Furthermore, the utilization of 2D cine mode enables gating strategies to detect and compensate for intrafractional motion. In addition, a recent multi-institutional Phase 2 trial on SMART demonstrated encouraging overall survival outcomes paired with an acceptable toxicity profile [11,12]. Interestingly, the study protocol allowed treating physicians to include a clinical target volume (CTV) to address potential microscopic disease alongside the gross tumor volume - a strategy that was adopted in most cases [13]. For several upper abdominal malignancies, SMART has already demonstrated dosimetric advantages, such as improved PTV coverage and reduced OAR constraint violations [14,15,16,17]. However, in these studies, only the gross tumor volume (GTV) was targeted, and no CTV or elective target volumes were considered. Therefore, in this analysis, we aimed to assess the benefits of SMART in patients with pancreatic cancer, with a specific focus on enhancing PTV coverage and reducing luminal gastrointestinal OAR dose exposure when elective low-dose volumes are included.

## Methods

### Patients and radiotherapy concept

Data of consecutive PDAC patients who underwent SMART at the University Hospital LMU in Munich between January 2020 and September 2024 were retrospectively analysed. Treatment indications were approved by an interdisciplinary tumor board and SBRT was performed in five fractions. All patients included in the analysis were part of a prospective observational study approved by the local ethics committee (LMU20-291).

### Baseline-plan generation

Patients were instructed to fast for 3 h prior to simulation and each treatment session. Treatment planning was performed using a 0.35 T hybrid MR-Linac system (MRIdian, ViewRay Inc., USA), as previously described [17,18]. Simulation included breath-hold MRI (True fast imaging with steady state precession (TRUFI) sequences) and a co-registered planning CT for electron density mapping. Breath-hold was performed following a breathing command, either in inspiration or expiration, depending on which position the patient could maintain most consistently. Target volumes and OARs were contoured on MRI using additional information from diagnostic imaging, following current guidelines [19], [10].

GTV and a CTV covering adjacent lymph node areas and vascular and neural tract regions were delineated on the simulation MRI. The CTV − PTV margin was 5 mm. Initially, a concept with a single dose level to the whole volume (tumor and elective volume) was applied; however, from 2022 onwards, a risk-adapted two-level approach was predominantly used, incorporating a low-dose elective target volume (PTV_elect) and a simultaneous integrated boost (SIB; PTV_SIB). Luminal gastrointestinal OARs (stomach, duodenum, bowel) were expanded by 3 mm to form PRVs. The overlap of these PRVs with the PTVs was subsequently subtracted from the PTVs to define an optimized PTV for dose optimization (PTV__OPT_ for patients treated with a single dose level and PTV_elect__OPT_ and PTV_SIB__OPT_, respectively, for patients treated with two dose levels).

A baseline treatment plan (PLAN_BASELINE_) was generated using inverse-planned step-and-shoot intensity-modulated radiation therapy with a 6 MV flattening filter-free beam and 1.5 mm isotropic grid resolution. Monte Carlo dose calculation was employed with a 1.0 % uncertainty level. The planning objectives were to achieve coverage of at least 95 % of the PTV__OPT_ / PTV_elect__OPT_ / PTV_SIB__OPT_ with at least 95 % of the prescribed, with a maximum dose (D_max_) of 125 % of the prescription. Constraints for luminal OARs were that the volume receiving 33 Gy or more (V_33Gy_) should be ≤ 0.5 cc for stomach, duodenum and small bowel loops.

### Plan adaptation and online reoptimization

For each fraction, a setup MRI scan was performed using the same TRUFI sequences as in simulation and was registered with the simulation MRI using deformable image registration. Contours and electron density were propagated, with manual adjustment of target volumes and OARs within the area of 3 cm of the PTV. Thereafter, the PLAN_BASELINE_ was recalculated on the treatment day's anatomy, resulting in a so-called PLAN_PREDICT_ for each fraction. If constraints were violated or coverage was inadequate, full online reoptimization was performed, resulting in PLAN_REOPTIMIZED_ (see figure A3 for a representative example). All reoptimized plans underwent secondary Monte Carlo verification before delivery.

During dose administration, the treatment incorporated a continuous real-time 2D Cine TRUFI sequence in a single sagittal plane to monitor and control the motion of the target volume and OARs. This involved establishing a gating boundary contour by adding a 3 mm isotropic margin to the GTV. Beam delivery was automatically paused if > 3–5 % of the GTV exceeded this gating boundary. In addition, motion management was supported by treating patients in breath-hold, maintaining the same breathing phase as used during simulation.

### Quantitative and categorial assessment of plan adaptation and follow-up

For the dosimetric assessment, data from all PDAC patients treated at our institution were collected from the MRIdian treatment planning system. Patients who underwent re-irradiation (n = 3) were excluded from the analysis due to differing margin and dose prescription strategies influenced by prior radiation therapy. The fulfilment of coverage prescription of PTV__OPT_ or PTV_SIB__OPT_ and PTV_elect__OPT_, respectively, and the fulfilment of dose constraints for the luminal gastrointestinal OAR structures were evaluated.

The categorical assessment of SMART benefit was evaluated for each patient and each fraction and classified into one of the following three categories:

(1) “useful” was defined when the planning aims were not fulfilled in the PLAN_PREDICT_, but were either fully met by the PLAN_REOPTIMIZED_ or when there was at least a 10 % improvement in PTV__OPT_ / PTV_SIB__OPT_ coverage and/or a reduction of OAR V_33Gy_ by at least 10 %.

(2) “futile” was defined when the PLAN_PREDICT_ violated planning aims, but PLAN_REOPTIMIZED_ failed to fully meet or substantially improve upon these objectives, as delineated above.

(3) “not necessary” was defined if the PLAN_PREDICT_ already fulfilled all planning aims and adaptation did not result in any substantial improvement.

During routine follow-up visits every three months, acute and late toxicities potentially related to radiotherapy were graded according to CTCAE v5.0 as part of standard clinical practice. Follow-up included a structured medical history covering the preceding three months, with specific attention to SBRT-related toxicities such as gastrointestinal bleeding, abdominal pain, obstructive symptoms, or cholangitis.

### Statistical analysis

In order to facilitate more effective comparison, the achieved dose values of PTV__OPT_, PTV_SIB__OPT_ and PTV_elect__OPT_ were normalized to the initial prescription for PTV coverage for each individual patient. For patients treated with one dose level, PTV__OPT_ was analysed. For patients with a two-dose level concept, PTV_elect__OPT_ and PTV_SIB__OPT_ were analysed. Acute and late toxicity were graded per CTCAE v5.0, with late toxicity defined as events occurring ≥ 90 days post-SMART. Statistical analysis was conducted using Microsoft Excel 365 (Microsoft Corporation; Redmond, WA, USA) and SPSS Version 29 (IBM Corp., Armonk, NY, USA) and Python Version 3.13.3 (Python Software Foundation; Wilmington, DE, USA). Coverage and OAR sparing of PLAN_PREDICT_ and PLAN_REOPTIMIZED_ were compared using paired t-tests. The influence of GTV and CTV size, dichotomized at the median, on PTV coverage improvement and OAR dose reduction was assessed with two-sided t-tests. Statistical significance was defined as p < 0.05.

## Results

### Patient and treatment characteristics

The patient and treatment characteristics are summarized in table 1. A total of 62 patients received SMART across 310 treatment fractions. Median age was 68.5 years (range: 43–89), with 60 % being male. Indications included metastatic (40 %), locally advanced (27 %), or locally recurrent (24 %) disease, with five patients (8 %) deemed medically inoperable. Among patients with metastatic disease, SMART was used for consolidation following chemotherapy, local control in progression, palliation, or in cases of chemotherapy intolerance. Most patients (69 %) had received prior systemic therapy. The pancreatic head was the most common tumor site (44 %).

All patients received treatment in five fractions. In 34 % of patients, 40 Gy in a single dose level was delivered to the macroscopic tumor and adjacent lymphatic and vascular/neural regions. In 19 %, the same areas were treated in palliative intent with 33 Gy. In 47 %, a two-level approach was used with 33 Gy to the elective volume and a 40 Gy SIB to the tumor. Median GTV was 37 cc (range: 7–162 cc). For single dose level plans, median PTV__OPT_ was 216 cc (range: 39–673 cc). For two dose level plans, median PTV_elect__OPT_ and PTV_SIB__OPT_ volumes were 210 cc (range: 132–662 cc) and 57 cc (range: 18–142 cc), respectively. Median treatment duration per fraction was 70 min (range 38–131 min, interquartile range 62–80 min), including patient positioning, image acquisition and registration, recontouring, plan adaptation, and treatment delivery. No acute grade ≥ 3 toxicity was detected. Late grade ≥ 3 events occurred in 5 patients (8 %): common bile duct stenosis (n = 1), gastric bleeding (n = 1), duodenal bleeding (grade 4, n = 2), and duodenal stenosis (n = 1). All were identified during symptom-triggered, unscheduled evaluations between 3 and 6 months post-MRIgRT.

### Evaluation of PLAN_BASELINE_

**Fig. A1:**
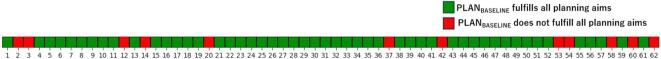
Fulfilment of planning aims of Plan_BASELINE_

Dose constraints were applied to the adjacent OARs. In all cases, dose constraints were required for the bowel due to the proximity of the PTVs to this organ. However, duodenum and stomach were not in close proximity to the PTVs in all cases. Thus, for duodenum and stomach, constraints were only applied in 45 patients (225 fractions) and 60 patients (300 fractions), respectively. The PLAN_BASELINE_ were found to meet the planning objectives in all but twelve patients (83 %, see figure A1). The reasons for not achieving the planning objectives in these twelve patients were as follows:

In four patients treated with a single-dose level approach, the achieved PTV__OPT_ coverage was 73.0 %, 84.3 %, 90.0 %, and 92.0 %, respectively, all of which were below the defined planning aim. In addition, in six patients treated with a two-dose level concept, the PTV_SIB__OPT_ coverage did not meet the constraints with 94.9 %, 89.1 %, 93.3 %, 91.1 %, 93.2 % and 94.4 %, respectively. However, in all these six cases, the PTV_elect__OPT_ coverage met the prescribed planning aim. Furthermore, two patients showed a modest exceeding of the gastric V_33Gy_ constraint, with volumes of 0.81 cc and 0.7 cc, respectively. One of these patients also had a suboptimal duodenal V_33Gy_ of 0.99 cc.

### Quantitative comparison of PLAN_PREDICT_ and PLAN_REOPTIMIZED_.

**Fig. 1:**
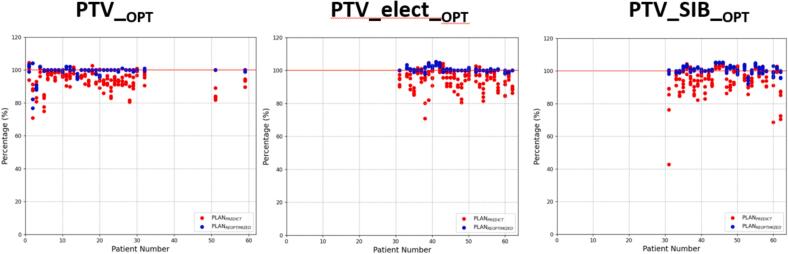
PTV coverage for non-adapted (PLAN_PREDICT_) and adapted (PLAN_REOPTIMIZED_) plans normalized to the coverage of the respective PTV in the baseline plan. Each dot shows the ratio of the calculated coverage to the baseline coverage. In some cases, the coverage was increased compared to the baseline plan, resulting in values > 100 %. A: PTV__OPT_; B: PTV_elect__OPT_; C: PTV_SIB__OPT._

Fig. 1 shows the comparison of the PTV coverage between PLAN_PREDICT_ and PLAN_REOPTIMIZED_. In patients treated with a single dose level, PTV__OPT_ was analysed. In patients with a two-dose level concept, PTV_elect__OPT_ and PTV_SIB__OPT_ were analysed.

The normalized PTV__OPT_ coverage did not meet clinical requirements and was < 95 % in 83/165 fractions (52 %) and < 90 % in 30 fractions (19 %) for the PLAN_PREDICT_. The PLAN_REOPTIMIZED_ improved coverage and resulted in a normalized coverage < 95 % in only 8 fractions (5.0 %) and < 90 % in 6 fractions (4 %). The PTV_SIB__OPT_ coverage normalized by the prescribed coverage was comparable with a coverage of < 95 % in 67/145 fractions (45 %) and < 90 % in 28 fractions (19 %) for the PLAN_PREDICT_. The PLAN_REOPTIMIZED_ resulted in a normalized coverage < 95 % in 3 fractions (2 %) and < 90 % in 0 fractions. The normalized PTV_elect__OPT_ coverage was < 95 % in 60/145 fractions (41 %) and < 90 % in 32 fractions (21 %) for the PLAN_PREDICT_. The PLAN_REOPTIMIZED_ resulted in a normalized coverage < 95 % in 0 fractions. The median coverage in the reoptimized plan was significantly higher than in the predicted plan for PTV__OPT_ (95.0 % vs 90.4 %, p < 0.001), PTV_elect__OPT_ (95.0 % vs. 91.1 %, p < 0.001), and for PTV_SIB__OPT_ (95.6 % vs. 91.3 %, p < 0.001) (see Table 2).

**Table 2:** Comparison of PLAN_BASELINE_ (n = 62), PLAN_PREDICT_ (n = 310) and PLAN_REOPTIMIZED_ (n = 310); p-values for comparison of PLAN_PREDICT_ and PLAN_REOPTIMIZED._

	PLAN_BASELINE_ median (range)	PLAN_PREDICT_ median (range)	PLAN_REOPTIMIZED_ median (range)	p-Value
PTV__OPT_, coverage (%)	95.0 (73.0–100)	90.4 (67.3–99.5)	95.0 (73–100)	<0.001
PTV_elect__OPT_, coverage (%)	95.0 (95.0–100)	91.1 (67.4–100)	95.0 (93–100)	<0.001
PTV_SIB__OPT_, coverage (%)	96.3 (89.1–100)	91.3 (40.6–100)	95.6 (88–100)	<0.001
Stomach V_33Gy_ (cc)	0.04 (0–0.81)	0.27 (0–12.69)	0.01 (0–0.93)	<0.001
Duodenum V_33Gy_ (cc)	0.09 (0–0.99)	1.07 (0–19.22)	0.07 (0–0.62)	<0.001
Bowel V_33Gy_ (cc)	0.11 (0–0.50)	2.04 (0–41.42)	0.06 (0–0.73)	<0.001

**Fig. 2:**
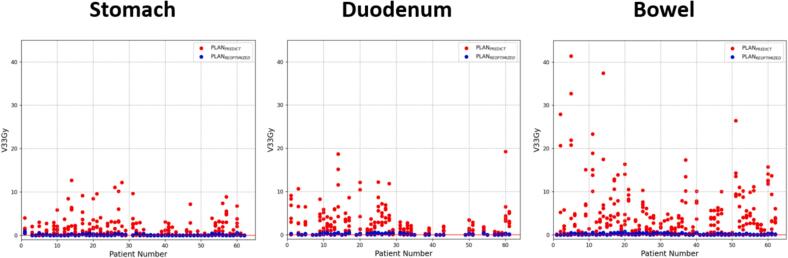
Compliance with dose constraints for non-adapted (PLAN_PREDICT_) and adapted (PLAN_REOPTIMIZED_) plans. The red horizontal line marks the institutional constraints of V_33Gy_ ≤ 0.5 cc.

The effect of SMART on meeting luminal gastrointestinal OAR constraints is depicted in Fig. 2. PLAN_PREDICT_ violated the constraint for the stomach in 129/300 fractions (43 %), for the duodenum in 138.

/225 fractions (61 %) and for the bowel in 216/310 fractions (70 %). In contrast, the Plan_REOPTIMIZED_ met the constraints for the stomach, duodenum and bowel in 99 %, 99 % and 98 %, respectively. The median predicted V_33Gy_ to the duodenum and bowel were 1.07 cc and 2.04 cc, respectively, exceeding the institutional constraint of 0.5 cc. After adaptation, the median V_33Gy_ values for the duodenum and bowel were significantly reduced to 0.07 cc and 0.06 cc, respectively, with both comparisons showing statistical significance (p < 0.001) (see Table 2). The median V_33Gy_ to the stomach in the PLAN_PREDICT_ was 0.27 cc, thus, below our institutional dose constraint of 0.5 cc. Nevertheless, the Plan_REOPTIMIZED_ resulted in a significant reduction to 0.01 cc (p < 0.001).

**Fig. A2:**
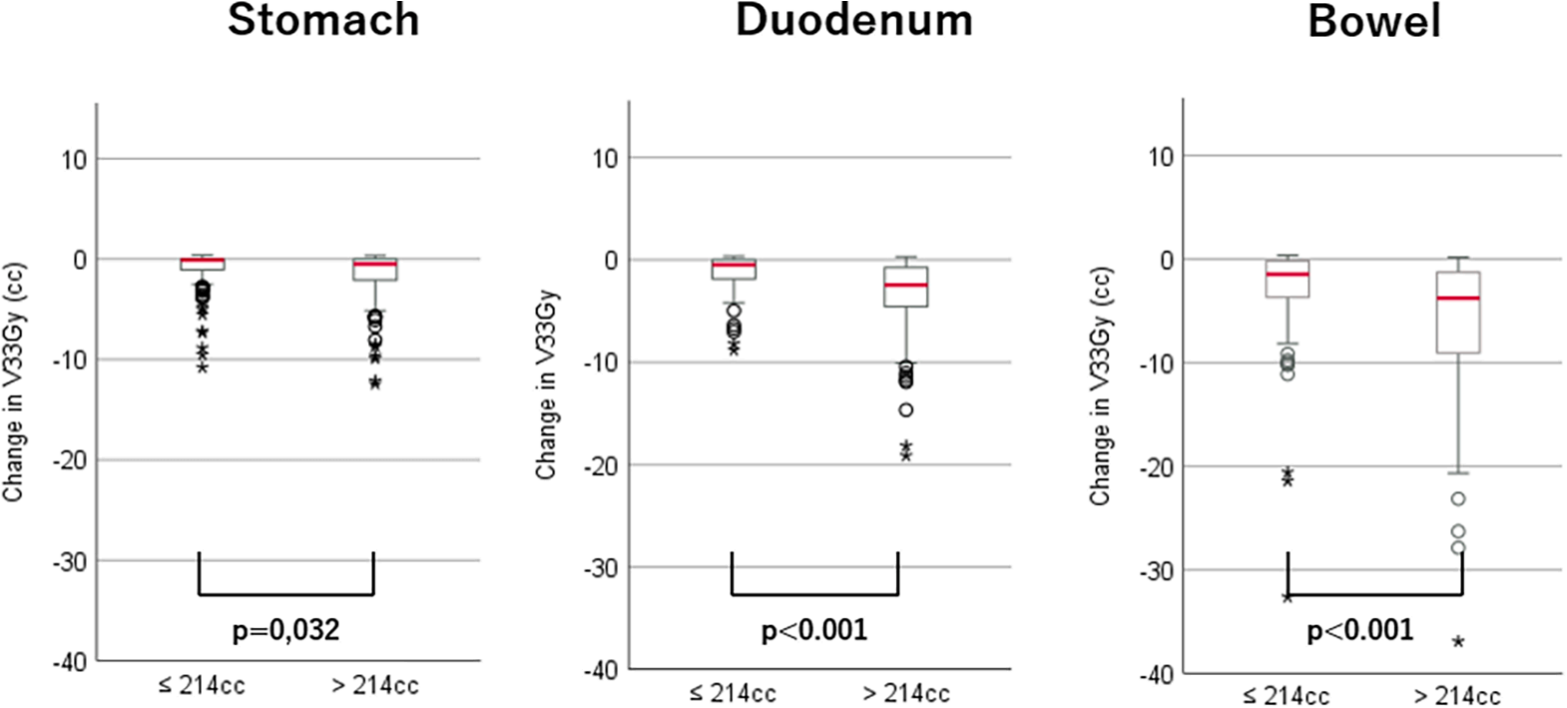
Comparison of the absolute improvement in OAR sparing between patients with a CTV ≤ 214 cc and > 214 cc.

**Fig. A3:**
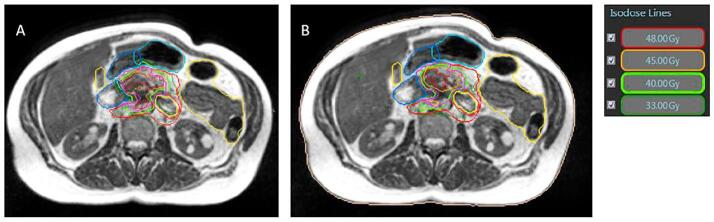
Representative axial slice of a treatment plan. Panel A shows the non-adapted plan (Plan_PREDICT_) with the 40  Gy isodose extending into the stomach (light blue) and duodenum (dark blue), along with suboptimal coverage of the PTV_elect__OPT_ (red) and the PTV_SIB__OPT_ (magenta). Panel B shows the adapted plan (Plan_REOPTIMIZED_) with improved sparing of OARs and more conformal PTV coverage. The small bowel is delineated in yellow.

The results of the t-tests assessing the influence of GTV and CTV volume size on OAR sparing and improvements in PTV coverage are summarized in Table A1 in the Appendix. A significantly greater improvement in OAR sparing with adaptive treatment was observed in patients with a CTV > 214 cc (Appendix, Fig. A2). Furthermore, these patients exhibited a significantly higher relative gain in PTV_elect__OPT_ coverage compared to those with smaller CTV volumes. In patients with a GTV > 37 cc, the absolute reduction in bowel V_33Gy_ was significantly more pronounced than in those with smaller GTVs. In contrast, GTV size had no significant impact on the sparing of the remaining two OARs or on overall improvement in PTV coverage.

**Fig. 3:**
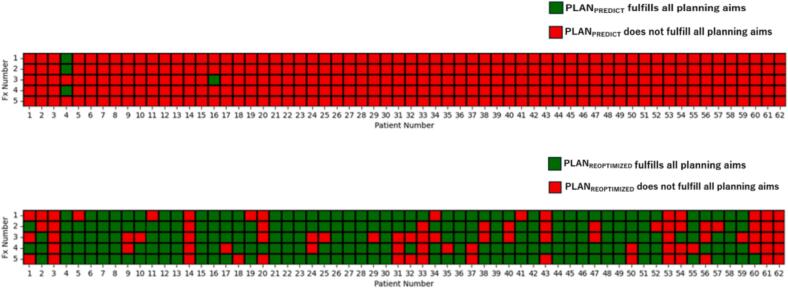
Fulfilment of planning aims of PLAN_PREDICT_ and PLAN_REOPTIMIZED._

Overall, the PLAN_PREDICT_ met all planning aims in only 4 out of 310 fractions (1 %) (see Fig. 3). In contrast, the PLAN_REOPTIMIZED_ met all planning objectives in 222 fractions (72 %).

### Categorial evaluation of plan adaptation

**Fig. 4:**
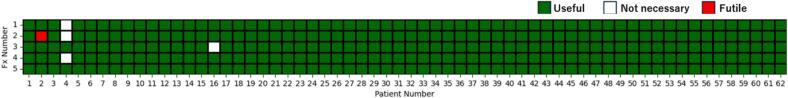
Categorial evaluation of online plan adaptation. The benefit of adaptation was classified as “useful”, “unnecessary”, or “futile” based on the criteria described in the Methods section.

The results of the categorial assessment are shown in Fig. 4. Based on the criteria described in the Methods section, online plan adaptation was useful in 305 / 310 fractions (98.4 %). It was not necessary in four fractions (1.3 %) and futile in one fraction (0.3 %). In 85 fractions (27 %), plan adaptation was categorized as useful due to an improvement in coverage or a reduction in V_33Gy_ by ≥ 10 %, even though not all planning aims were met.

## Discussion

In this large dosimetric series of 310 SMART fractions for PDAC, PLAN_BASELINE_ met all planning objectives in 83 % of patients. In the remaining cases the most common reason for not meeting the objectives was a suboptimal PTV coverage, which was tolerated to prioritize our primary goal of optimal OAR sparing. A modest exceedance of the institutional V_33Gy_ OAR constraint was accepted in only two patients. However, volumes remained below 1 cc, which is regarded as an acceptable V_33Gy_ constraint in several other publications [14], [20], [21], [22]. Furthermore, with all plans undergoing daily adaptation, we prioritized generating robust initial plans over fully optimizing plans that would not be delivered as originally conceived.

Although some degree of unmet planning objectives was expected due to the mobility of upper abdominal organs and results of other dosimetric studies on SMART for PDAC, it was surprising that the PLAN_PREDICT_ would have met all planning objectives in only 3 % of the fractions [14], [23], [24]. Without adaptation, at least one luminal gastrointestinal OAR constraint would have been exceeded in 58 % of fractions, with predicted V_33Gy_ values for the stomach, duodenum, and bowel going up to a maximum of 13 cc, 19 cc, and 41 cc, respectively. With online adaptation, nearly three-quarters of the fractions met all planning objectives. In the remaining fractions, the most common reason for not meeting the objectives was again suboptimal PTV coverage, which was accepted due to our priority of OAR sparing. In no fraction did the PLAN_REOPTIMIZED_ exceed a V_33Gy_ of 1 cc. These results underscore the interfractional uncertainty associated with SBRT in the upper abdomen and highlights the importance of online adaptation.

Of note, patients with ≥ 4 fractions exceeding planning aims in PLAN_REOPTIMIZED_ shared several features. First, all had either a CTV or GTV above the cohort median (CTV > 214 cc or GTV > 37 cc), leading to larger PTVs and greater overlap with adjacent luminal OARs. Second, in 8 of 10 cases, tumors were located in the pancreatic head or represented local recurrences, where anatomical constraints inherently limited sparing potential. Third, all were treated with a SIB and large elective volumes, further challenging consistent constraint adherence despite reoptimization.

Importantly, the most significant constraint exceedance in the PLAN_PREDICT_, both in terms of maximum and median values, was observed for the bowel. This finding is particularly relevant because other studies on SMART primarily reported constraint exceedance for the duodenum and stomach, while bowel constraints were met in the vast majority of fractions [14], [23]. The primary reason for this discrepancy - in addition to an unequal distribution of tumor locations - is that in these studies, only the GTV was treated with a PTV margin, resulting in smaller target volumes that were not located near bowel structures. For example, in the study by Bohoudi et al., the PTV_OPT_ was created using a 3 mm margin around the GTV while actively excluding luminal OAR. No elective volume was included. Consequently, in their analysis, the predicted plan exceeded the V_33Gy_ constraints in only < 2 %, 13 %, and 29 % of fractions for the bowel, stomach, and duodenum, respectively. In contrast, our study incorporated an elective treatment volume in addition to the macroscopic tumor, leading to larger target volumes and, consequently, higher rates of predicted constraint exceedance for the bowel (70 % of fractions), stomach (43 %), and duodenum (61 %). This elective target volume strategy is supported by recurrence-mapping and surgical “Triangle” data highlighting perivascular and neural relapse sites [25], [26], [27], [28], [29], [30], [31], as well as radiation series showing improved outcomes with elective nodal irradiation [26], [32], [33], [34]. In a recent multi-institutional Phase 2 trial, the study protocol allowed physicians to use a CTV at their discretion, resulting in 54 % of patients being treated with an elective CTV [11]. They reported violations of gastroluminal OAR in 96 % of fractions as the primary reason for plan adaptation, with specific violations of duodenum, stomach, small bowel, and large bowel in 79 %, 65 %, 47 %, and 26 % of fractions, respectively. However, level 1 evidence supporting the inclusion of elective treatment volumes remains lacking, and their use remains a topic of debate in current guidelines [19], [10].

PTV coverage was acceptable in several fractions already with the PLAN_PREDICT_, and online adaptation yielded smaller relative gains in PTV coverage than in OAR sparing. This reflects the fact that positional and morphological changes of the tumor between fractions were less pronounced compared to the highly mobile and filling-dependent gastrointestinal organs. However, it still prevented target coverage from decreasing to below 90 % in nearly half the fractions. For all three evaluated luminal OARs, the absolute reduction in V_33Gy_ was significantly greater in patients with a larger CTV. As expected, elective CTV size also had a significant impact on the relative improvement in PTV_elect__OPT_ coverage achieved through online adaptation. In contrast, GTV size was only associated with a significant absolute dose reduction for the bowel V_33Gy_. This finding can be explained by the target volume concepts: when elective target volumes are included, it is primarily the size of the CTV-rather than the GTV-that determines the extent of benefit from SMART.

Since full compliance with planning aims is not the only quality criterion for adaptation, a categorial evaluation was also introduced, wherein any marked improvement (defined as ≥ 10 %) was also considered useful. In our study, SMART provided a benefit in almost all cases (>98 %). This contrasts with the study by Bohoudi et al., which categorized the effects of SMART into three similar groups and observed a benefit in only 53 % of cases [14]. Online adaptation was particularly beneficial for patients with a distance between the GTV and adjacent OARs of < 3 mm. This is likely due to the previously mentioned differences in target volume generation, which led to smaller target volumes in their study compared to ours (median PTV_OPT_ 48 cc vs 218 cc). In our study, the CTV of the elective volumes was < 3 mm from at least one OAR in all cases. Therefore, our analysis highlights that such close proximity is almost inevitable when elective target volumes are included, underscoring that SMART is particularly relevant in this setting. This is also reflected in the low rate of higher-grade toxicity, which in all cases, was deemed to be potentially rather than definitely attributable to SMART and which is comparable to other studies and series on SMART [12], [13].

In addition to SMART, cone-beam computed tomography-guided stereotactic adaptive radiotherapy (CT-STAR) has emerged as another online adaptive treatment approach. However, data for CT-STAR in PDAC are limited, consisting mainly of in silico studies and small case series [35], [36], [37], [38], [39]. Early findings indicate that adaptation with CT-STAR also improves OAR sparing and PTV coverage, similar to the results observed in our study. Moreover, the commercially available CT-STAR system on the Varian Ethos platform offers AI-assisted online delineation, which can reduce workflow resource demands. For instance, Lee et al. reported an average treatment time of 29 min, compared to a median treatment time of 70 min in our study [39]. However, another report from an institution with access to both SMART and CT-STAR systems found that treatment times were comparable between the two approaches [40]. For the comparison of the two techniques, in addition to potential time savings, it is important to consider that SMART provides enhanced soft-tissue contrast and enables intrafraction motion monitoring through 2D cine imaging. However, data on a direct comparison are not yet available.

Limitations of the study include the relatively small sample size. However, among the dosimetric studies on SMART, this study includes the largest patient cohort to date. Furthermore, the dosimetric analysis was performed on a per-fraction basis, which may not fully capture cumulative effects such as compensation for OAR overdoses or PTV under-coverage over the entire five-fraction treatment course. Moreover, minor changes in target volumes over the treatment course cannot be entirely excluded and may have influenced the usefulness classification in isolated cases; however, this effect is likely negligible given the predominant role of OAR sparing in our analysis. Additionally, the interval between image acquisition and treatment delivery represents a potential source of intrafractional uncertainty, which could slightly affect plan quality, particularly for OARs whose position or volume may change during treatment. Finally, our study focuses exclusively on treatments involving irradiation including elective target volumes. There is currently no Phase I evidence comparing this approach to SBRT targeting only the GTV. Nevertheless, this can also be seen as a strength, as detailed dosimetric analyses on this topic have been lacking so far. Moreover, the use of elective target volumes is already being implemented in ongoing studies and is also reflected in current guidelines [11], [19].

## Conclusion

For the vast majority of PDAC patients treated with stereotactic radiotherapy with elective target volumes daily online adaptation was of benefit improving target coverage and OAR sparing, supporting its broader adoption in high-precision pancreatic radiotherapy.

### Patient consent

The author(s) confirm that written informed consent has been obtained from the involved patient(s) or if appropriate from the parent, guardian, power of attorney of the involved patient(s); and, they have given approval for this information to be published in this case report (series).

## Declaration of competing interest

The authors declare that they have no known competing financial interests or personal relationships that could have appeared to influence the work reported in this paper.

## Figures and Tables

**Table A1:** Results of the *t*-test comparing the influence of GTV size (≤37 cc vs > 37 cc) and CTV size (≤214 cc vs > 214 cc) on the relative improvement of PTV coverage and absolute change of OAR V_33Gy_ through SMART.

	GTV ≤ 37 cc	GTV > 37 cc	p-Value	CTV ≤ 214 cc	CTV > 214 cc	p-Value
Relative improvement PTV__OPT_ coverage, mean (%)	−7.3	−7.6	0.704	7.5	6.0	0.142
Relative improvement PTV_elect__OPT_ coverage, mean (%)	−5.8	−7.8	0.076	8.2	5.1	**0.005**
Relative improvement PTV_SIB__OPT_ coverage, mean (%)	−6.8	−6.4	0.904	6.5	7.8	0.672
Absolute change of stomach V_33Gy_ (cc)	−1.3	−1.1	0.689	−1.0	1.6	**0.032**
Absolute change of duodenum V_33Gy_ (cc)	−1.7	−2.4	0.081	−1.3	−3.3	**<0.001**
Absolute change of bowel V_33Gy_ (cc)	−3.0	−5.2	**<0.001**	−3.0	−5.8	**<0.001**
